# Supplementary material for: Machine learning assisted differentiation of low acuity patients at dispatch: The MADLAD randomized controlled trial
Source: PLoS Med. 2026 Mar 31;23(3):e1004770. doi: 10.1371/journal.pmed.1004770 (PMC13037975; doi:10.1371/journal.pmed.1004770)
Supplement: S1 Supplementary Material — 1. Check missingness. 2. Perform multiple imputation. 3. Evaluate multiple imputation. 4. Baseline data. 5. Primary hypothesis. 6. Secondary Hypothesis 1 - Outcome measure. 7. Secondary Hypotheses 2 - NEWS. difference. 8. Evaluate using OLS. 9. Density plot of news differences. 10. Ancillary analyses. 10.1) Evaluation of overall personnel compliance with the risk assessment instrument in intervention arm. a) Per-model assignment accuracy (post hoc). 10.2) Evaluation of compliance in intervention arm cases where the model had a high versus low level of confidence. a) Confidence groups with per-model assignment (post hoc). 10.3) Evaluation of improved/degraded compliance with risk assessment instrument over time as manifested by a slope change in a time series analysis of intervention group. 10.4) Evaluation of spillover effects as manifested by a significant positive slope in a time series analysis of control group outcomes. 10.5) Evaluation of change in risk assessment tool predictive value over time (covariate drift). 10.6) Evaluation of model calibration with regards to age, gender, and complaint category. 11. Post-randomization loss to follow-up. 12. Descriptive statistics of cases excluded due to patient refusals. 13. Evaluation of study arm imbalance. 14. Simulate accuracy with random assignment. 15. Additional post-hoc analyses. a) Model versus Dispatcher preference analysis. b) Estimate accuracy using model assignments in high-confidence intervention cases. c) Parameter summary/variable importance. d) AUC in retrospective test and trial data. (HTML) [file pmed.1004770.s001.html]

Supplement 1 - Analysis notebook


# Supplement 1 - Analysis notebook

- Check
  missingness
- Perform multiple
  imputation
- Evaluate multiple
  imputation
- Baseline
  data
- Primary
  hypothesis
- Secondary Hypothesis 1
  - Outcome measure
- Secondary Hypotheses 2
  - NEWS difference
  - Evaluate
    using OLS
  - Density plot of news
    differences
- Ancillary
  analyses
  - 1.
    Evaluation of overall personnel compliance with risk assessment
    instrument in intervention arm.
    - Per-model assignment
      accuracy (post hoc)
  - 2.
    Evaluation of compliance in intervention arm cases where the model had a
    high vs low level of confidence.
    - Confidence
      groups with per-model assignment (post hoc)
  - 3.
    Evaluation of improved/degraded compliance with risk assessment
    instrument over time as manifested by a slope change in a time series
    analysis of intervention group
  - 4.
    Evaluation of spillover effects as manifested by a significant positive
    slope in a time series analysis of control group outcomes
  - 5.
    Evaluation of change in risk assessment tool predictive value over time
    (covariate drift)
  - 6.
    Evaluation of model calibration with regards to age, gender, and
    complaint category
  - Post-randomization loss to
    follow-up
    - Descriptive
      statistics of cases excluded due to patient refusals
    - Evaluation of study arm
      imbalance
- Simulate accuracy with
  random assignment
- Additional post-hoc analyses
  - Model vs Dispatcher
    preference analysis
  - Estimate
    accuracy using model assignments in high-confidence intervention
    cases
  - Parameter summary /
    variable importance
  - AUC in retrospective
    test and trial data

This supplement details the statistical procedures used in the
article “Machine Learning Assisted Differentiation of Low Acuity
Patients at Dispatch (MADLAD): A Randomized Clinical Trial”.

```
library(tidyverse)
library(mice)
library(knitr)
library(rjson)
library(DT)
library(pROC)

options(scipen = 999)

set.seed(42)


ci_bootstrap <- function(x, 
                         fun,
                         conf_level = 0.95, 
                         n_bootstrap = 1000,
                         na.rm = T,
                         print = F,
                         pct = F,
                         r=1) {
  
  # Generate ordinary bootstrap estimates of CI for descriptive stats
  # Note: Use only on original data, not multiple imputations!
  
  est = fun(x,na.rm = na.rm)
  
  boots <- replicate(n_bootstrap, {
    sample_x <- sample(x, 
                       length(x), 
                       replace = TRUE)
    fun(sample_x,na.rm = na.rm)
  })
  lower_bound <- quantile(boots, 
                          (1 - conf_level) / 2)
  upper_bound <- quantile(boots, 
                          1 - (1 - conf_level) / 2)
  if(pct){
    est = est*100
    lower_bound = lower_bound*100
    upper_bound = upper_bound*100
  }
  
  if(print){
    return(paste0(round(est,r)," (",round(lower_bound,r),"-",round(upper_bound,r),")"))
  }else{
    return(c(est,lower_bound, upper_bound))
  }
  
}


news_calc <- function(data){
  
  # Calculate a NEWS score from vital signs
  # From https://www.rcplondon.ac.uk/file/9434/download?token=kf8WbPib
  
  out <- data %>%
    transmute(news_rr = ifelse(amb_vital_breaths <= 8, 3,
                               ifelse(amb_vital_breaths <= 11,1,
                                      ifelse(amb_vital_breaths <= 20,0,
                                             ifelse(amb_vital_breaths <= 24,2, 3)))),
              # We'll use scale 1 of the NEWS scoring chart
              news_spo2 = ifelse(amb_vital_spo2 <= 91, 3,
                                 ifelse(amb_vital_spo2 <= 93,2,
                                        ifelse(amb_vital_spo2 <= 95,1, 0))),
              news_o2 = ifelse(amb_o2 > 0,2,0),
              news_sbp = ifelse(amb_vital_bpsys <= 90, 3,
                                ifelse(amb_vital_bpsys <= 100,2,
                                       ifelse(amb_vital_bpsys <= 110,1,
                                              ifelse(amb_vital_bpsys <= 219,0, 3)))),
              news_pr = ifelse(amb_vital_pulse <= 40, 3,
                               ifelse(amb_vital_pulse <= 50,1,
                                      ifelse(amb_vital_pulse <= 90,0,
                                             ifelse(amb_vital_pulse <= 110,1,
                                                    ifelse(amb_vital_pulse <= 130,2, 3))))),
              
              news_con = ifelse(amb_conscious == 0,0,3),
              
              news_temp = ifelse(amb_vital_temp <= 35, 3,
                                 ifelse(amb_vital_temp <= 36,1,
                                        ifelse(amb_vital_temp <= 38,0,
                                               ifelse(amb_vital_temp <= 39,1, 2))))) %>%
    mutate(news_full = rowSums(dplyr::select(., starts_with("news_"))))
  return(out)
}

# Map category names to english
names_map = c("Allergisk reaktion" = "Allergic Reaction", 
  "Allmän barn" = "General Child", 
  "Allmän vuxen" = "General Adult", 
  "Allmän åldring" = "General Elderly", 
  "Andningsbesvär" = "Difficulty Breathing", 
  "Annat" = "Other", 
  "Arm-, bensymtom (ej trauma)" = "Arm/leg sympoms (non-traumatic)", 
  "Blod i urin" = "Blood in urine", 
  "Blodig upphostning" = "Blood in cough", 
  "Blodsocker lågt" = "Low Blood sugar", 
  "Brand" = "Fire", 
  "Brännskada" = "Burn", 
  "Bröstsmärta" = "Chest pain", 
  "Buk-, flanksmärta" = "Abdominal/flank pain", 
  "CBRN" = "CBRN", 
  "Diarré" = "Diarrhea", 
  "Drunkningstillbud" = "Drowning", 
  "Dykeriolycka" = "Diving accident", 
  "Elektrisk skada" = "Electical injury", 
  "Feber" = "Fever", 
  "Flygolycka" = "Aircraft accident", 
  "Förlossning" = "Childbirth", 
  "Förvirring" = "Confision", 
  "Graviditet" = "Pregnancy", 
  "Hallucination" = "Hallucination", 
  "Halsont" = "Sore throat", 
  "Hjärtstopp" = "Cardiac arrest", 
  "Huvudvärk" = "Headache", 
  "Hypotermi" = "Hypothermia", 
  "ICD" = "ICD", 
  "Illamående" = "Nausea", 
  "Infektion" = "Infection", 
  "Intox/förgiftning" = "Intoxication", 
  "Kramper" = "Convulsions", 
  "Kräkning" = "Vomiting", 
  "Köldskada" = "Cold injury", 
  "Luftvägsbesvär" = "Airway problems", 
  "Mag-, tarmblödning" = "Stomach/Intestinal bleeding", 
  "Näs-, svalgblödning" = "Nose/Throat bleeding", 
  "Ormbett" = "Snake bite", 
  "Pacemaker" = "Pacemaker", 
  "Planerad" = "Planned",
  "Psykiska besvär" = "Psychiatic problems", 
  "Ryggsmärta" = "Back pain", 
  "Rytmrubbning" = "Cardiac arrythmia", 
  "Rökexponering" = "Smoke exposure", 
  "Saknas" = "MBS Unused", 
  "Sensoriskt-, motoriskt bortfall" = "Senosory/motor deficiency", 
  "Sjöolycka" = "Maritime accident", 
  "Stroke" = "Stroke", 
  "Svimning" = "Fainting", 
  "Sårskada" = "Minor truama", 
  "Sänkt vakenhet" = "Reduced Consciousness", 
  "Trafikolycka" = "Traffic accident", 
  "Trauma" = "Trauma", 
  "Urinkateterstopp" = "Urinary catheter blockage", 
  "Urinstämma" = "Urinary retention", 
  "Urogenitala besvär" = "Urogenital problems", 
  "Vaginal blödning" = "Vaginal bleeding", 
  "Våld-hot-suicidhot" = "Violence/threats/Suicide", 
  "Yrsel" = "Dizziness", 
  "Ögon" = "Eye problems")

names(names_map) <- make.names(names(names_map))
```

```
# Load trial data
trial_data <- read_csv("Study/data/241218_trial_data.csv") %>%
  mutate(first_assigned = ifelse(is.na(first_assigned),F,first_assigned),
         assign_delay = as.numeric(assign_time - incl_time),
         age = year(created_time)- as.numeric(substr(pid,1,4)),
         female = as.numeric(substr(pid,11,11)) %% 2,
         hour = hour(created_time))
```

```
## Rows: 4207 Columns: 24
## ── Column specification ────────────────────────────────────────────────────────
## Delimiter: ","
## chr  (9): Group, conf, Text, TrialID, region, closeReason, closeDetail, clos...
## dbl  (8): id, Score, trial_cases, max_score, prio_out, pid, first_assigned_i...
## lgl  (3): excl_patient, first_assigned, excl_multi_test
## dttm (4): incl_time, created_time, assign_time, arrive_time
## 
## ℹ Use `spec()` to retrieve the full column specification for this data.
## ℹ Specify the column types or set `show_col_types = FALSE` to quiet this message.
```

```
# Load ambulance/hospital outcome data

pred_data <- read.csv("Study/data/241223_pred_data.csv")
```

```
# apply inclusion criteria

incl_data <- trial_data %>%
  group_by(TrialID) %>%
  filter(!any(excl_patient),
         !excl_multi_test,
         any(!is.na(assign_time))
  ) %>%
  ungroup() 

# Generate dataset for multiple imputation

mi_data <- select(incl_data,id,TrialID,max_score,Group,first_assigned,assign_delay) %>%
  left_join(pred_data) %>%
  mutate(amb_cat_avpu = as.factor(amb_cat_avpu),
         hosp_vital_avpu = as.factor(hosp_vital_avpu),
         region = as.factor(region),
         closeDetail = as.factor(closeDetail),
         closeDestination = as.factor(closeDestination),
         # Remove two obviously misdocumented pulse values of 1022 and 8670
         amb_vital_pulse = ifelse(amb_vital_pulse>500,
                                  NA,amb_vital_pulse)) %>%
  # Calculate NEWS for complete data
  bind_cols(news_calc(.)) %>%
  #Colinear with NEWS components
  select(-amb_o2,
         -amb_conscious)
```

```
## Joining with `by = join_by(id)`
```

## Check missingness

```
missingness <- mi_data  %>%
  mutate(any_vital_conscious = ifelse(is.na(news_con),hosp_vital_avpu,news_con),
         any_vital_o2 = ifelse(is.na(news_o2),hosp_oxygen,news_o2),
         any_vital_bpsys = ifelse(is.na(amb_vital_bpsys),hosp_vital_bpsys,amb_vital_bpsys),
         any_vital_pulse = ifelse(is.na(amb_vital_pulse),hosp_vital_pulse,amb_vital_pulse),
         any_vital_breaths = ifelse(is.na(amb_vital_breaths),hosp_vital_breaths,amb_vital_breaths),
         any_vital_spo2 = ifelse(is.na(amb_vital_spo2),hosp_vital_spo2,amb_vital_spo2),
         any_vital_temp = ifelse(is.na(amb_vital_temp),hosp_vital_temp,amb_vital_temp)) %>%
  select(starts_with("amb_v"), starts_with("hosp_v"), starts_with("any_v"),-hosp_vital_avpu) %>%
  pivot_longer(everything()) %>%
  group_by(name) %>%
  summarise(mean = mean(value,na.rm=T),
            miss = mean(is.na(value)))

kable(missingness,
      caption = "Missingness rates overall")
```

Missingness rates overall

| name | mean | miss |
| --- | --- | --- |
| amb\_vital\_bpsys | 135.4111882 | 0.1139653 |
| amb\_vital\_breaths | 20.0761274 | 0.1255427 |
| amb\_vital\_delay | -855.2400413 | 0.1671491 |
| amb\_vital\_gcs | 14.8129176 | 0.1877713 |
| amb\_vital\_pulse | 86.4485563 | 0.1103473 |
| amb\_vital\_rls | 1.0250000 | 0.9131693 |
| amb\_vital\_spo2 | 95.6010531 | 0.1067294 |
| amb\_vital\_temp | 37.0747786 | 0.1421852 |
| any\_vital\_bpsys | 135.5469875 | 0.0452243 |
| any\_vital\_breaths | 19.9100569 | 0.0466715 |
| any\_vital\_conscious | 0.1954887 | 0.0376266 |
| any\_vital\_o2 | 0.1789316 | 0.0314761 |
| any\_vital\_pulse | 86.3128070 | 0.0423300 |
| any\_vital\_spo2 | 95.7461218 | 0.0437771 |
| any\_vital\_temp | 37.0565019 | 0.0484805 |
| hosp\_vital\_bpsys | 135.8199390 | 0.2887120 |
| hosp\_vital\_breaths | 18.9574689 | 0.3024602 |
| hosp\_vital\_diabp | 75.7894737 | 0.9931259 |
| hosp\_vital\_pulse | 83.9064565 | 0.2883502 |
| hosp\_vital\_spo2 | 96.5404848 | 0.2984805 |
| hosp\_vital\_temp | 36.9559640 | 0.2963097 |

```
missingness_per_region <- mi_data  %>%
  mutate(any_vital_conscious = ifelse(is.na(news_con),hosp_vital_avpu,news_con),
         any_vital_o2 = ifelse(is.na(news_o2),hosp_oxygen,news_o2),
         any_vital_bpsys = ifelse(is.na(amb_vital_bpsys),hosp_vital_bpsys,amb_vital_bpsys),
         any_vital_pulse = ifelse(is.na(amb_vital_pulse),hosp_vital_pulse,amb_vital_pulse),
         any_vital_breaths = ifelse(is.na(amb_vital_breaths),hosp_vital_breaths,amb_vital_breaths),
         any_vital_spo2 = ifelse(is.na(amb_vital_spo2),hosp_vital_spo2,amb_vital_spo2),
         any_vital_temp = ifelse(is.na(amb_vital_temp),hosp_vital_temp,amb_vital_temp)) %>%
  select(region, starts_with("amb_v"), starts_with("hosp_v"), starts_with("any_v"),-hosp_vital_avpu) %>%
  pivot_longer(-region) %>%
  group_by(name,region) %>%
  summarise(mean = mean(value,na.rm=T),
            miss = mean(is.na(value)))
```

```
## `summarise()` has grouped output by 'name'. You can override using the
## `.groups` argument.
```

```
kable(missingness_per_region,
      caption = "Missingness rates per region")
```

Missingness rates per region

| name | region | mean | miss |
| --- | --- | --- | --- |
| amb\_vital\_bpsys | Uppsala | 135.4990749 | 0.1200651 |
| amb\_vital\_bpsys | Vastmanland | 134.7491289 | 0.0651466 |
| amb\_vital\_breaths | Uppsala | 20.2245186 | 0.1334961 |
| amb\_vital\_breaths | Vastmanland | 18.9791667 | 0.0618893 |
| amb\_vital\_delay | Uppsala | -679.8480089 | 0.1798942 |
| amb\_vital\_delay | Vastmanland | -2086.6510000 | 0.0651466 |
| amb\_vital\_gcs | Uppsala | 14.8010674 | 0.1611722 |
| amb\_vital\_gcs | Vastmanland | 14.9456522 | 0.4006515 |
| amb\_vital\_pulse | Uppsala | 86.5094427 | 0.1164021 |
| amb\_vital\_pulse | Vastmanland | 85.9895833 | 0.0618893 |
| amb\_vital\_rls | Uppsala | NaN | 1.0000000 |
| amb\_vital\_rls | Vastmanland | 1.0250000 | 0.2182410 |
| amb\_vital\_spo2 | Uppsala | 95.4745530 | 0.1123321 |
| amb\_vital\_spo2 | Vastmanland | 96.5590278 | 0.0618893 |
| amb\_vital\_temp | Uppsala | 37.0821103 | 0.1514042 |
| amb\_vital\_temp | Vastmanland | 37.0213287 | 0.0684039 |
| any\_vital\_bpsys | Uppsala | 135.6779770 | 0.0463980 |
| any\_vital\_bpsys | Vastmanland | 134.5101351 | 0.0358306 |
| any\_vital\_breaths | Uppsala | 20.0226593 | 0.0480260 |
| any\_vital\_breaths | Vastmanland | 19.0202703 | 0.0358306 |
| any\_vital\_conscious | Uppsala | 0.2037272 | 0.0390720 |
| any\_vital\_conscious | Vastmanland | 0.1304348 | 0.0260586 |
| any\_vital\_o2 | Uppsala | 0.1854500 | 0.0321530 |
| any\_vital\_o2 | Vastmanland | 0.1270903 | 0.0260586 |
| any\_vital\_pulse | Uppsala | 86.3276523 | 0.0447700 |
| any\_vital\_pulse | Vastmanland | 86.1966667 | 0.0228013 |
| any\_vital\_spo2 | Uppsala | 95.6474605 | 0.0463980 |
| any\_vital\_spo2 | Vastmanland | 96.5166667 | 0.0228013 |
| any\_vital\_temp | Uppsala | 37.0606775 | 0.0508751 |
| any\_vital\_temp | Vastmanland | 37.0238255 | 0.0293160 |
| hosp\_vital\_bpsys | Uppsala | 135.8258860 | 0.2075702 |
| hosp\_vital\_bpsys | Vastmanland | 135.2105263 | 0.9381107 |
| hosp\_vital\_breaths | Uppsala | 18.9523311 | 0.2230362 |
| hosp\_vital\_breaths | Vastmanland | 19.4736842 | 0.9381107 |
| hosp\_vital\_diabp | Uppsala | NaN | 1.0000000 |
| hosp\_vital\_diabp | Vastmanland | 75.7894737 | 0.9381107 |
| hosp\_vital\_pulse | Uppsala | 83.8642674 | 0.2083842 |
| hosp\_vital\_pulse | Vastmanland | 87.6363636 | 0.9283388 |
| hosp\_vital\_spo2 | Uppsala | 96.5477308 | 0.2197802 |
| hosp\_vital\_spo2 | Vastmanland | 95.9090909 | 0.9283388 |
| hosp\_vital\_temp | Uppsala | 36.9545762 | 0.2173382 |
| hosp\_vital\_temp | Vastmanland | 37.0772727 | 0.9283388 |

## Perform multiple imputation

```
init <- mice(mi_data,
             maxit = 0,
             method="rf")
```

```
## Warning: Number of logged events: 5
```

```
pred <- init$pred

# Don't include administrative trial data in the imputation
pred[,c("id","TrialID","max_score","Group","first_assigned","assign_delay")] <- 0
pred[c("id","TrialID","max_score","Group","first_assigned","assign_delay"),] <- 0

meth <- init$method

meth["assign_delay"] <- ""


if(file.exists("mi_obj.rda")){
  load("mi_obj.rda")
}else{
  mi_obj <- mice(mi_data,maxit = 15,m = 10, pred = pred,method=meth)

  save(mi_obj,file = "mi_obj.rda")
}

mi_long_full <- complete(mi_obj,action = "long", include = TRUE) %>%
  # Doing item level imputation and calculating scores directly per https://www.tandfonline.com/doi/abs/10.1080/00273171.2012.640589
  select(-news_full) %>%
  mutate(news_full = rowSums(dplyr::select(., starts_with("news_")))) %>%
  mutate(out_primary = pmin(pmax(amb_airway,amb_breathing,amb_circulation,news_con),1),
         out_prio = pmin(pmax(amb_prio,amb_crit),1),
         out_intervention = pmin(pmax(amb_meds,amb_iv,news_o2,amb_alert),1),
         out_hosp = pmin(pmax(hosp_admit,hosp_30daymort),1),
         out_composite = out_primary*4+out_prio*2+out_intervention+out_hosp) %>%
  # comment this to analyze real scores ----
  # mutate(news_full = ifelse(is.na(news_full),NA,
  #                           rpois(nrow(.),3)),
  # mutate(out_composite = ifelse(is.na(out_composite),NA,
  #                           rpois(nrow(.),2))) %>%
  # --------------------------------------
  group_by(.imp,TrialID) %>%
  mutate(first_assigned_pp = ifelse(Group == "intervention",
                                    as.logical(max_score),first_assigned),
         max_news = news_full == max(news_full),
         news_diff = news_full[first_assigned] - mean(news_full[!first_assigned],na.rm=T),
         news_diff_pp = news_full[first_assigned_pp] - mean(news_full[!first_assigned_pp],na.rm=T),
         max_out = out_composite == max(out_composite,na.rm = T)) %>%
  ungroup()
```

```
## Warning: There were 181 warnings in `mutate()`.
## The first warning was:
## ℹ In argument: `max_out = out_composite == max(out_composite, na.rm = T)`.
## ℹ In group 4: `.imp = 0` and `TrialID = "20210202195449_775475_775522_ap0"`.
## Caused by warning in `max()`:
## ! no non-missing arguments to max; returning -Inf
## ℹ Run `dplyr::last_dplyr_warnings()` to see the 180 remaining warnings.
```

```
mi_long <- mi_long_full %>%
  #Analyze based on properties of first assigned patient
  filter(as.logical(first_assigned)) %>%
  left_join(select(incl_data,id,TrialID,Score,conf),by = c("id","TrialID")) %>%
  mutate(conf = relevel(factor(conf),ref = "low"),
         date = ymd(substr(TrialID,1,8)),
         study_month = (date - min(date))/30) %>%
  select(-starts_with("cat"),-starts_with("mbs"))

#Some warnings due to missing values in original data
#Logged events are due to constants: No patients recieved CPR, and the dataset contains only first-assigned patients
mi_test <- mi_long %>% as.mids()
```

```
## Warning: Number of logged events: 2
```

```
mi_test_ua <- mi_long %>% filter(region == "Uppsala") %>% as.mids()
```

```
## Warning: Number of logged events: 3
```

## Evaluate multiple imputation

```
plot(mi_obj,layout = c(2,39),main = "Multiple imputation chain mixing diagnostic plot")
```

# Baseline data

```
table1 <- incl_data %>%
  left_join(filter(mi_long_full,.imp == 1)) %>%
  group_by(Group) %>%
  summarise(n_rcs = as.character(length(unique(TrialID))),
            n_patient= as.character(n()),
            median_age = ci_bootstrap(age,median,print=T),
            pct_female = ci_bootstrap(female,mean,print=T,pct=T),
            median_wait = ci_bootstrap(assign_time - created_time,median,na.rm=T,print=T),
            median_wait_first = ci_bootstrap(assign_time[first_assigned] - created_time[first_assigned],
                                             median,na.rm=T,print=T),
            median_assign_delay_first = ci_bootstrap(assign_delay[first_assigned],median,na.rm=T,print=T),
            mean_news = ci_bootstrap(news_full,mean,print=T)
  ) %>%
  pivot_longer(-Group) %>%
  pivot_wider(names_from = Group)
```

```
## Joining with `by = join_by(id, Group, TrialID, max_score, region, closeDetail,
## closeDestination, first_assigned, assign_delay, age, female, hour)`
```

```
kable(table1,
      caption = "Baseline data")
```

Baseline data

| name | control | intervention |
| --- | --- | --- |
| n\_rcs | 585 | 660 |
| n\_patient | 1285 | 1479 |
| median\_age | 77 (76-78) | 77 (76-78) |
| pct\_female | 45.4 (42.6-48.2) | 43.7 (41.3-46.2) |
| median\_wait | 45.1 (42.7-48.6) | 44.6 (42-46.8) |
| median\_wait\_first | 32.1 (30.4-35.5) | 29.2 (26.5-31) |
| median\_assign\_delay\_first | 202 (176.1-254.7) | 95.6 (81.5-132.9) |
| mean\_news | 3 (2.8-3.3) | 2.8 (2.6-3) |

# Primary hypothesis

```
mi_long %>%
  filter(.imp != 0) %>%
  group_by(Group) %>%
  summarise(mean(max_news))
```

```
fit_mi_prim <- with(mi_test,
                    glm(max_news ~ Group,
                        family = "binomial"))


test_PH <- summary(pool(fit_mi_prim),conf.int = TRUE)

select(test_PH, term) %>%
  bind_cols(exp(select(test_PH,estimate,`2.5 %`,`97.5 %`))) %>%
kable(.,caption = "Primary hypothesis coefficients")
```

Primary hypothesis coefficients

| term | estimate | 2.5 % | 97.5 % |
| --- | --- | --- | --- |
| (Intercept) | 1.670101 | 1.404096 | 1.986500 |
| Groupintervention | 1.282307 | 1.003256 | 1.638976 |

# Secondary Hypothesis 1 - Outcome measure

```
mi_long %>%
  filter(.imp != 0) %>%
  group_by(Group) %>%
  summarise(mean(max_out))
```

```
fit_mi_sec_out <- with(mi_test_ua,
                    glm(max_out ~ Group,
                        family = "binomial"))


test_SH1 <- summary(pool(fit_mi_sec_out),conf.int = T)

select(test_SH1, term) %>%
  bind_cols(exp(select(test_SH1,estimate,`2.5 %`,`97.5 %`))) %>%
kable(.,caption = "Secondary hypothesis 1 coefficients")
```

Secondary hypothesis 1 coefficients

| term | estimate | 2.5 % | 97.5 % |
| --- | --- | --- | --- |
| (Intercept) | 1.990586 | 1.648567 | 2.403563 |
| Groupintervention | 1.318519 | 1.010762 | 1.719984 |

# Secondary Hypotheses 2 - NEWS difference

```
# Function to perform Wilcoxon test on a single imputed dataset
wilcoxon_test <- function(data) {
  result <- wilcox.test(news_diff ~ Group, data = data)
  z <- qnorm(1 - result$p.value / 2) # Transform p-value to z-score
  return(as.numeric(z)) # Ensure the result is numeric
}

# Apply the Wilcoxon test to each imputed dataset
z_scores <- sapply(1:mi_test$m, function(i) {
  imputed_dataset <- complete(mi_test, i)
  wilcoxon_test(imputed_dataset)
})

# Pool z-scores using Rubin's rules
pooled_z <- mean(z_scores)
var_within <- var(z_scores) / length(z_scores)
pooled_var <- var_within
pooled_p <- 1.96 * (1 - pnorm(abs(pooled_z)))


cat("Secondary hypothesis 2 Pooled z-score:", pooled_z)
```

```
## Secondary hypothesis 2 Pooled z-score: 1.71588
```

```
cat("Secondary hypothesis 2 Pooled p-value:", pooled_p)
```

```
## Secondary hypothesis 2 Pooled p-value: 0.08446032
```

### Evaluate using OLS

```
filter(mi_long,.imp != 0) %>% 
  group_by(Group) %>% 
  summarise(pct_max_news = mean(max_news),
            mean_news = mean(news_diff)) %>%
  kable(caption ="Average NEWS across groups")
```

Average NEWS across groups

| Group | pct\_max\_news | mean\_news |
| --- | --- | --- |
| control | 0.6254701 | 0.6220228 |
| intervention | 0.6816667 | 1.0761995 |

```
fit_mi_sec_diff <- with(mi_test,
                       lm(news_diff ~ Group))


test_SH2 <- summary(pool(fit_mi_sec_diff),conf.int = T)

kable(test_SH2,
      caption = "Secondary hypothesis 2 evaluated using OLS")
```

Secondary hypothesis 2 evaluated using OLS


| term | estimate | std.error | statistic | df | p.value | 2.5 % | 97.5 % |
| --- | --- | --- | --- | --- | --- | --- | --- |
| (Intercept) | 0.5832479 | 0.1648130 | 3.538846 | 1104.72 | 0.0004186 | 0.2598660 | 0.9066298 |
| Groupintervention | 0.4541132 | 0.2256152 | 2.012778 | 1154.11 | 0.0443699 | 0.0114514 | 0.8967751 |

### Density plot of news differences

```
ggplot(aes(x=news_diff,
           group = paste(Group),
           color=paste(Group),
           fill=paste(Group)),
       data = filter(mi_long,.imp != 0)) +
  geom_density(bw=2,alpha = 0.5) +
  labs(title = "Density plot of NEWS differences in control and intervention groups")
```

# Ancillary analyses

## 1. Evaluation of overall personnel compliance with risk assessment instrument in intervention arm.

```
# Checking both arms to provide a basis for comparison
mi_long %>%
  filter(.imp == 0) %>%
  group_by(Group) %>%
  summarise(compliance = ci_bootstrap(max_score,fun = mean,print=T,r=3)) %>%
  kable(caption = "Agreement rate with risk assessment tool across study arms")
```

Agreement rate with risk assessment tool across study
arms

| Group | compliance |
| --- | --- |
| control | 0.54 (0.501-0.579) |
| intervention | 0.809 (0.779-0.839) |

### Per-model assignment accuracy (post hoc)

```
mi_long_pp <- mi_long_full %>%
  filter(first_assigned_pp) %>%
  left_join(select(incl_data,id,TrialID,Score,conf),by = c("id","TrialID")) %>%
  mutate(conf = relevel(factor(conf),ref = "low"))

mi_test_pp <- mi_long_pp %>% as.mids()
```

```
## Warning: Number of logged events: 5
```

```
filter(mi_long_pp,.imp != 0) %>% 
  group_by(Group) %>% 
  summarise(pct_max_news = mean(max_news),
            mean_news = mean(news_diff_pp)) %>%
  kable(caption ="Average NEWS across groups")
```

Average NEWS across groups

| Group | pct\_max\_news | mean\_news |
| --- | --- | --- |
| control | 0.6254701 | 0.6220228 |
| intervention | 0.7221212 | 1.5856692 |

```
ggplot(aes(x=news_diff_pp,
           group = paste(Group),
           color=paste(Group),
           fill=paste(Group)),
       data = filter(mi_long_pp,.imp != 0)) +
  geom_density(bw=2,alpha = 0.5) +
  labs(title = "Density plot of NEWS differences in control and intervention groups assuming full compliance")
```

```
fit_mi_prim_pp <- with(mi_test_pp,
                    glm(max_news ~ Group,
                        family = "binomial"))

test_add1 <- summary(pool(fit_mi_prim_pp),conf.int = TRUE)


select(test_add1, term) %>%
  bind_cols(exp(select(test_add1,estimate,`2.5 %`,`97.5 %`))) %>%
kable(.,caption = "Primary hypothesis coefficients with full compliance in intervention group")
```

Primary hypothesis coefficients with full compliance in
intervention group

| term | estimate | 2.5 % | 97.5 % |
| --- | --- | --- | --- |
| (Intercept) | 1.670101 | 1.404096 | 1.986500 |
| Groupintervention | 1.556207 | 1.210694 | 2.000323 |

## 2. Evaluation of compliance in intervention arm cases where the model had a high vs low level of confidence.

```
mi_long %>%
  filter(.imp == 0) %>%
  group_by(Group,conf) %>%
  summarise(compliance = ci_bootstrap(max_score,fun = mean,print=T,r=3)) %>%
  kable(caption = "Compliance across confidence groups")
```

```
## `summarise()` has grouped output by 'Group'. You can override using the
## `.groups` argument.
```

Compliance across confidence groups

| Group | conf | compliance |
| --- | --- | --- |
| control | NA | 0.54 (0.501-0.581) |
| intervention | low | 0.803 (0.762-0.843) |
| intervention | high | 0.817 (0.772-0.862) |

```
fit_mi_prim_conf <- with(filter(mi_test,Group == "intervention"),
                            glm(max_news ~ conf,
                                family = "binomial"))


test_add2 <- summary(pool(fit_mi_prim_conf),conf.int = TRUE)


select(test_add2, term) %>%
  bind_cols(exp(select(test_add2,estimate,`2.5 %`,`97.5 %`))) %>%
kable(.,caption = "Primary hypothesis difference between confidence groups")
```

Primary hypothesis difference between confidence
groups

| term | estimate | 2.5 % | 97.5 % |
| --- | --- | --- | --- |
| (Intercept) | 2.038423 | 1.6068566 | 2.585900 |
| confhigh | 1.120809 | 0.7830795 | 1.604195 |

### Confidence groups with per-model assignment (post hoc)

```
mi_long_pp %>%
  filter(.imp != 0) %>%
  group_by(Group,conf) %>%
  summarise(pct_max_news = mean(max_news),
            mean_news_difference = mean(news_diff_pp))
```

```
## `summarise()` has grouped output by 'Group'. You can override using the
## `.groups` argument.
```

```
# Density plot of news differences with confidence groups
ggplot(aes(x=news_diff_pp,
           group = paste(Group,conf),
           color=paste(Group,conf),
           fill=paste(Group,conf)),
       data = filter(mi_long,.imp != 0)) +
  geom_density(bw=2,alpha = 0.5) +
  labs(title = "Density plot of NEWS differences in control and\nhigh/low confidence intervention groups")
```

```
fit_mi_prim_conf_pp <- with(filter(mi_test_pp,Group == "intervention"),
                            glm(max_news ~ conf,
                                family = "binomial"))


test_add2_pp <- summary(pool(fit_mi_prim_conf_pp),conf.int = TRUE)


select(test_add2_pp, term) %>%
  bind_cols(exp(select(test_add2_pp,estimate,`2.5 %`,`97.5 %`))) %>%
kable(.,caption = "Primary hypothesis difference between confidence groups with full compliance")
```

Primary hypothesis difference between confidence groups with
full compliance

| term | estimate | 2.5 % | 97.5 % |
| --- | --- | --- | --- |
| (Intercept) | 2.099143 | 1.671775 | 2.635762 |
| confhigh | 1.682797 | 1.168247 | 2.423980 |

## 3. Evaluation of improved/degraded compliance with risk assessment instrument over time as manifested by a slope change in a time series analysis of intervention group

```
mi_long %>%
  filter(Group == "intervention",.imp != 0) %>%
  group_by(month = floor_date(ymd(substr(TrialID,1,8)),unit = "months")) %>%
  summarise(pct_comply = mean(max_score)) %>%
  ggplot(aes(x=month,y=pct_comply)) +
  geom_line() +
  geom_smooth(method = "lm") +
  labs(title = "Time series of compliance rate with risk assessment instrument per month with linear trend")
```

```
## `geom_smooth()` using formula = 'y ~ x'
```

```
fit_mi_sec_compliance <- with(filter(mi_test,Group == "intervention"),
                              glm(max_score ~  study_month,
                                  family = "binomial"))

test_add3 <- summary(pool(fit_mi_sec_compliance),conf.int = TRUE)

select(test_add3, term) %>%
  bind_cols(exp(select(test_add3,estimate,`2.5 %`,`97.5 %`))) %>%
kable(.,caption = "Coefficient for compliance rates per month")
```

Coefficient for compliance rates per month

| term | estimate | 2.5 % | 97.5 % |
| --- | --- | --- | --- |
| (Intercept) | 5.8186614 | 3.866226 | 8.757072 |
| study\_month | 0.9876977 | 0.974348 | 1.001230 |

Some negative slope, but not significant

## 4. Evaluation of spillover effects as manifested by a significant positive slope in a time series analysis of control group outcomes

```
mi_long %>%
  filter(Group == "control",.imp != 0) %>%
  group_by(month = floor_date(ymd(substr(TrialID,1,8)),unit = "months")) %>%
  summarise(pct_correct = mean(max_news)) %>%
  ggplot(aes(x=month,y=pct_correct)) +
  geom_line() +
  geom_smooth(method = "lm") +
  labs(title = "Time series of proportion of correct assessments in control group per month with linear trend")
```

```
## `geom_smooth()` using formula = 'y ~ x'
```

```
fit_mi_sec_spillover <- with(filter(mi_test,Group == "control"),
                             glm(max_news ~  study_month,
                                 family = "binomial"))


test_add4 <- summary(pool(fit_mi_sec_spillover),conf.int = TRUE)

select(test_add4, term) %>%
  bind_cols(exp(select(test_add4,estimate,`2.5 %`,`97.5 %`))) %>%
kable(.,caption = "Coefficient for proportion of correct assessments in control group per month")
```

Coefficient for proportion of correct assessments in control
group per month

| term | estimate | 2.5 % | 97.5 % |
| --- | --- | --- | --- |
| (Intercept) | 1.650823 | 1.1833415 | 2.302986 |
| study\_month | 1.000496 | 0.9883983 | 1.012741 |

No significant effects

## 5. Evaluation of change in risk assessment tool predictive value over time (covariate drift)

```
update_1 <- ymd("20211201")
update_2 <- ymd("20240601")

mi_long %>%
  filter(.imp != 0) %>%
  group_by(month = floor_date(ymd(substr(TrialID,1,8)),unit = "months")) %>%
  summarise(score_news_corr = cor(Score,news_full, method = "spearman")) %>%
  ggplot(aes(x=month,y=score_news_corr)) +
  geom_line() +
  geom_vline(xintercept = update_1,color = "red") +
  geom_vline(xintercept = update_2,color = "red") +
  labs(title = "Time series of correlation bewteen model prediction and NEWS scores per month")
```

```
mi_ts <- mi_long %>%
  transmute(date = floor_date(ymd(substr(TrialID,1,8)),unit = "months"),
            news_full,
            Score,
            .imp,
            .id) %>%
  group_by(date,.imp) %>%
  summarise(score_news_cor = cor(Score,news_full,method = "spearman",use = "complete.obs")) %>%
  group_by(.imp) %>%
  mutate(.id = row_number()) %>%
  mutate(study_date = as.numeric(date - min(date)),
         update_1_date = ifelse(as.numeric(date - update_1)>0,as.numeric(date - update_1),100000),
         update_2_date = ifelse(as.numeric(date - update_2)>0,as.numeric(date - update_2),100000),
         since_update = pmin(study_date,update_1_date,update_2_date)/30) %>%
  as.mids()
```

```
## `summarise()` has grouped output by 'date'. You can override using the
## `.groups` argument.
```

```
fit_mi_sec_predvalue <- with(mi_ts,
                             lm(score_news_cor ~  since_update))


test_add5 <- summary(pool(fit_mi_sec_predvalue),conf.int = TRUE)

select(test_add5, term) %>%
  bind_cols(exp(select(test_add5,estimate,`2.5 %`,`97.5 %`))) %>%
kable(.,caption = "Coefficient for correlation bewteen model prediction and NEWS scores per month")
```

Coefficient for correlation bewteen model prediction and NEWS
scores per month

| term | estimate | 2.5 % | 97.5 % |
| --- | --- | --- | --- |
| (Intercept) | 1.5693886 | 1.4332750 | 1.718428 |
| since\_update | 0.9996419 | 0.9935602 | 1.005761 |

No significant effects

## 6. Evaluation of model calibration with regards to age, gender, and complaint category

```
cats <- select(pred_data,id,starts_with("cat")) %>%
  pivot_longer(-id) %>%
  filter(value == 2) %>%
  mutate(name = gsub("cat.","",name))

# Including all cases here, not just first assigned to investigate scores
mi_long_calib <- mi_long_full %>%
  left_join(select(incl_data,id,TrialID,Score,conf)) %>%
  left_join(cats) %>%
  mutate(name = plyr::revalue(name,names_map))
```

```
## Joining with `by = join_by(id, TrialID)`
## Joining with `by = join_by(id)`
## The following `from` values were not present in `x`: Annat, Brand, CBRN,
## Drunkningstillbud, Dykeriolycka, Elektrisk.skada, Flygolycka, Förlossning,
## Graviditet, Hjärtstopp, Hypotermi, ICD, Köldskada, Ormbett, Pacemaker,
## Planerad, Rökexponering, Saknas, Sjöolycka, Trafikolycka, Våld.hot.suicidhot
```

```
mi_test_calib <- mi_long_calib %>% as.mids()
```

```
## Warning: Number of logged events: 4
```

```
fit_mi_sec_calib_age <- with(mi_test_calib,
                             lm(news_full ~  Score+age))

test_add6_age <- summary(pool(fit_mi_sec_calib_age),conf.int = TRUE)

test_add6_age %>%
  select(term) %>%
  bind_cols(exp(select(test_add6_age,estimate,`2.5 %`,`97.5 %`))) %>%
  kable(caption = "Coefficints for age adjusted by score")
```

Coefficints for age adjusted by score

| term | estimate | 2.5 % | 97.5 % |
| --- | --- | --- | --- |
| (Intercept) | 8.039782 | 5.4212203 | 11.923162 |
| Score | 7.415745 | 6.3264673 | 8.692572 |
| age | 1.003647 | 0.9982391 | 1.009084 |

```
fit_mi_sec_calib_female <- with(mi_test_calib,
                             lm(news_full ~  Score+female))

test_add6_female <- summary(pool(fit_mi_sec_calib_female),conf.int = TRUE)

test_add6_female %>%
  select(term) %>%
  bind_cols(exp(select(test_add6_female,estimate,`2.5 %`,`97.5 %`))) %>%
  kable(caption = "Coefficints for gender adjusted by score")
```

Coefficints for gender adjusted by score

| term | estimate | 2.5 % | 97.5 % |
| --- | --- | --- | --- |
| (Intercept) | 11.0298281 | 9.668094 | 12.583360 |
| Score | 7.7721917 | 6.686645 | 9.033971 |
| female | 0.8752795 | 0.718316 | 1.066542 |

```
fit_mi_sec_calib_category <- with(mi_test_calib,
                             lm(news_full ~  Score+name))

test_add6_cat <- summary(pool(fit_mi_sec_calib_category),conf.int = TRUE)

test_add6_cat %>%
  select(term) %>%
  bind_cols(exp(select(test_add6_cat,estimate,`2.5 %`,`97.5 %`))) %>%
  kable(caption = "Coefficints for call types adjusted by score")
```

Coefficints for call types adjusted by score

| term | estimate | 2.5 % | 97.5 % |
| --- | --- | --- | --- |
| (Intercept) | 7.1586440 | 5.2421279 | 9.775836 |
| Score | 4.4687164 | 3.6145851 | 5.524680 |
| nameAirway problems | 0.8222224 | 0.1705789 | 3.963266 |
| nameAllergic Reaction | 0.5534450 | 0.1094782 | 2.797831 |
| nameArm/leg sympoms (non-traumatic) | 2.3075855 | 1.1952943 | 4.454928 |
| nameBack pain | 1.2462212 | 0.6472364 | 2.399536 |
| nameBlodsocker.högt. | 0.5713130 | 0.0945157 | 3.453378 |
| nameBlood in cough | 0.1886438 | 0.0012787 | 27.829299 |
| nameBlood in urine | 1.1756227 | 0.2928085 | 4.720111 |
| nameBurn | 0.3912147 | 0.0026728 | 57.261566 |
| nameCardiac arrythmia | 0.5779412 | 0.2812316 | 1.187690 |
| nameChest pain | 0.9336384 | 0.4582425 | 1.902226 |
| nameConfision | 0.7201735 | 0.2731643 | 1.898674 |
| nameConvulsions | 0.5118968 | 0.1584104 | 1.654173 |
| nameDiarrhea | 1.0126907 | 0.2901245 | 3.534835 |
| nameDifficulty Breathing | 6.0719564 | 3.8798655 | 9.502560 |
| nameDizziness | 0.9285364 | 0.5569722 | 1.547977 |
| nameEye problems | 0.1517840 | 0.0006098 | 37.782364 |
| nameFainting | 0.7179065 | 0.3685947 | 1.398256 |
| nameFever | 5.1766164 | 2.5611842 | 10.462878 |
| nameGeneral Adult | 1.1867209 | 0.6195507 | 2.273109 |
| nameGeneral Child | 0.2021712 | 0.0010191 | 40.107552 |
| nameGeneral Elderly | 1.5766009 | 1.0009524 | 2.483305 |
| nameHallucination | 0.1449157 | 0.0034711 | 6.050065 |
| nameHeadache | 0.6680986 | 0.2273807 | 1.963032 |
| nameInfection | 2.6833481 | 1.5330629 | 4.696713 |
| nameIntoxication | 0.9935221 | 0.3859669 | 2.557437 |
| nameLow Blood sugar | 0.3017404 | 0.0371465 | 2.451036 |
| nameMinor truama | 0.8960731 | 0.1355553 | 5.923393 |
| nameNausea | 0.4531260 | 0.0972606 | 2.111063 |
| nameNose/Throat bleeding | 1.1230826 | 0.4116997 | 3.063676 |
| namePsychiatic problems | 0.4671649 | 0.1372096 | 1.590581 |
| nameReduced Consciousness | 6.6438112 | 0.3686053 | 119.749307 |
| nameSenosory/motor deficiency | 0.5182861 | 0.0659971 | 4.070189 |
| nameSore throat | 0.2410450 | 0.0016452 | 35.316289 |
| nameStomach/Intestinal bleeding | 1.6390336 | 0.5573864 | 4.819693 |
| nameStroke | 1.1715987 | 0.5930723 | 2.314462 |
| nameTrauma | 1.1334531 | 0.7851340 | 1.636302 |
| nameUrinary catheter blockage | 0.9021973 | 0.0880556 | 9.243709 |
| nameUrinary retention | 0.4202743 | 0.0901666 | 1.958935 |
| nameUrogenital problems | 3.7388744 | 0.3005881 | 46.506110 |
| nameVaginal bleeding | 0.4022629 | 0.0482959 | 3.350501 |
| nameVomiting | 0.8236679 | 0.3413326 | 1.987589 |

No significant effect of age/gender beyond model predictions, 2/41
categories significant (Breathing difficulty & fever), but
equivalent to the number of significant coefficients generated by
chance.

## Post-randomization loss to follow-up

```
excl_summ <- trial_data %>%
  group_by(TrialID,Group,first_assigned_id) %>%
  summarise(excl_patient = !any(excl_patient),
            excl_protocol = !all(excl_multi_test) & any(!is.na(assign_time))) %>%
  group_by(first_assigned_id) %>%
  arrange(TrialID) %>% #Note that the trial ID is initiated with a timestamp, so this orders the trials by inclusion time
  mutate(group_first = first(Group)) %>%
  ungroup()
```

```
## `summarise()` has grouped output by 'TrialID', 'Group'. You can override using
## the `.groups` argument.
```

```
# Number of randomizations per arm
table(excl_summ$Group)
```

```
## 
##      control intervention 
##          876          969
```

```
# Number of exclusions due to protocol violations
sum(!excl_summ$excl_protocol)
```

```
## [1] 350
```

```
#Number of exclusions due to patient refusals
sum(!excl_summ$excl_patient & excl_summ$excl_protocol)
```

```
## [1] 250
```

```
# Number of exclusions due to protocol violations per arm
sum(!excl_summ$excl_protocol[excl_summ$Group == "control"])
```

```
## [1] 175
```

```
sum(!excl_summ$excl_protocol[excl_summ$Group == "intervention"])
```

```
## [1] 175
```

```
#Number of exclusions due to patient refusals
sum(!excl_summ$excl_patient[excl_summ$Group == "control"] & excl_summ$excl_protocol[excl_summ$Group == "control"])
```

```
## [1] 116
```

```
sum(!excl_summ$excl_patient[excl_summ$Group == "intervention"] & excl_summ$excl_protocol[excl_summ$Group == "intervention"])
```

```
## [1] 134
```

### Descriptive statistics of cases excluded due to patient refusals

```
excl_data <- trial_data %>%
  group_by(TrialID) %>%
  mutate(excl_patient = !any(excl_patient),
         excl_protocol = !all(excl_multi_test) & any(!is.na(assign_time)))

## Descriptive data per table 1 for included and excluded calls
table1_excl <- excl_data %>%
  # Exclude protocol violations before analysis
  filter(excl_protocol) %>%
  group_by(excl_patient) %>%
  summarise(n_rcs = as.character(length(unique(TrialID))),
            n_patient= as.character(n()),
            median_age = ci_bootstrap(age,median,print=T),
            pct_female = ci_bootstrap(female,mean,print=T,r=2),
            median_wait = ci_bootstrap(assign_time - created_time,median,na.rm=T,print=T),
            median_wait_first = ci_bootstrap(assign_time[first_assigned] - created_time[first_assigned],median,na.rm=T,print=T),
            median_assign_delay_first = ci_bootstrap(assign_delay[first_assigned],median,na.rm=T,print=T),
            pct_agreement = ci_bootstrap(max_score == first_assigned,mean,na.rm=T,print=T,r=2)
  ) %>%
  pivot_longer(-excl_patient) %>%
  pivot_wider(names_from = excl_patient)

kable(table1_excl,
      caption = "Baseline data for excluded vs. included patients")
```

Baseline data for excluded vs. included patients

| name | FALSE | TRUE |
| --- | --- | --- |
| n\_rcs | 250 | 1245 |
| n\_patient | 589 | 2764 |
| median\_age | 77 (76-79) | 77 (76-78) |
| pct\_female | 0.42 (0.38-0.46) | 0.45 (0.43-0.46) |
| median\_wait | 46.6 (42.3-50.2) | 44.7 (43-46.7) |
| median\_wait\_first | 29.5 (23.8-33.5) | 30.7 (29.3-32.4) |
| median\_assign\_delay\_first | 151.9 (117.8-200.7) | 148.2 (133.7-176.3) |
| pct\_agreement | 0.71 (0.67-0.74) | 0.71 (0.7-0.73) |

### Evaluation of study arm imbalance

```
# Significance of study arm imbalance
prop.test(nrow(excl_summ[excl_summ$Group == "intervention",]),
          nrow(excl_summ))
```

```
## 
##  1-sample proportions test with continuity correction
## 
## data:  nrow(excl_summ[excl_summ$Group == "intervention", ]) out of nrow(excl_summ), null probability 0.5
## X-squared = 4.5875, df = 1, p-value = 0.03221
## alternative hypothesis: true p is not equal to 0.5
## 95 percent confidence interval:
##  0.5021175 0.5481830
## sample estimates:
##         p 
## 0.5252033
```

```
## Working hypothesis is that this is caused (at least in part) by dispatchers re-randomizing control cases, which would result in the first randomization in a series of trials excluded due to differing study arm allocation being in the control group:

first_group <- excl_summ %>%
  group_by(first_assigned_id) %>%
  filter(!excl_protocol) %>%
  ungroup() %>%
  summarise(n = n(),
            control = sum(group_first == "control"))


kable(first_group)
```

| n | control |
| --- | --- |
| 350 | 200 |

```
prop.test(first_group$control,first_group$n)
```

```
## 
##  1-sample proportions test with continuity correction
## 
## data:  first_group$control out of first_group$n, null probability 0.5
## X-squared = 6.86, df = 1, p-value = 0.008815
## alternative hypothesis: true p is not equal to 0.5
## 95 percent confidence interval:
##  0.5176504 0.6236133
## sample estimates:
##         p 
## 0.5714286
```

We see that the study arm imbalance is significant (p = 0.032). Of
the 350 total exclusions due to protocol violations, 200 were initially
in the control group, significantly more than could be expected by
chance (p= 0.009), lending credence to our suspicion that this could be
caused by dispatchers preferentially to re-randomizing control arm
RCS.

# Simulate accuracy with random assignment

```
for(i in 1:1000){

null_mi_long <- mi_long_calib %>%
  filter(.imp == 1) %>% # Single imputation
  group_by(TrialID) %>%
  mutate(first_assigned_null = sample(n()) == 1,
         news_diff = news_full[first_assigned_null] -
           mean(news_full[!first_assigned_null])) %>%
  filter(first_assigned_null)

if(i==1){
  null_decisions <- data_frame("r" = i,
                               "max_news" = mean(null_mi_long$max_news),
                               "max_out" = mean(null_mi_long$max_out),
                               "news_diff" = mean(null_mi_long$news_diff)
                               )
}else{
    null_decisions <- bind_rows(null_decisions,
                                data_frame("r" = i,
                                "max_news" = mean(null_mi_long$max_news),
                               "max_out" = mean(null_mi_long$max_out),
                               "news_diff" = mean(null_mi_long$news_diff)))
}


}
```

```
## Warning: `data_frame()` was deprecated in tibble 1.1.0.
## ℹ Please use `tibble()` instead.
## This warning is displayed once every 8 hours.
## Call `lifecycle::last_lifecycle_warnings()` to see where this warning was
## generated.
```

```
# Average proportion of correct news score assignmeds given random decisions
mean(null_decisions$max_news)
```

```
## [1] 0.5575052
```

```
# Average proportion of correct outcome score assignmens given random decisions
mean(null_decisions$max_out)
```

```
## [1] 0.5756747
```

```
# Average NEWS difference given random decisions
mean(null_decisions$news_diff)
```

```
## [1] 0.004326372
```

Across 1000 simulated trials, the accuracy of random assignement in
directing the first available ambulance to the patient with the highest
NEWS score was 55.7%

# Additional post-hoc analyses

## Model vs Dispatcher preference analysis

```
long_compliance <- mi_long_calib %>%
  filter(.imp == 1) # For this minor analysis, use single imputation

comply_table_dem <- long_compliance %>%
  group_by(Group,max_score,first_assigned) %>%
  summarise(n = n(),
            median_age = ci_bootstrap(age,median,print=T),
            pct_female = ci_bootstrap(female,mean,print=T,pct=T),
            mean_news = ci_bootstrap(news_full,mean,print=T))
```

```
## `summarise()` has grouped output by 'Group', 'max_score'. You can override
## using the `.groups` argument.
```

```
# Similar demographic characteristics across compliance groups
kable(comply_table_dem)
```

| Group | max\_score | first\_assigned | n | median\_age | pct\_female | mean\_news |
| --- | --- | --- | --- | --- | --- | --- |
| control | 0 | FALSE | 431 | 75 (73-77) | 38.1 (33.6-42.7) | 1.7 (1.5-2) |
| control | 0 | TRUE | 269 | 73 (71-75) | 42.8 (36.1-48.7) | 1.9 (1.7-2.2) |
| control | 1 | FALSE | 269 | 79 (77-80) | 56.9 (51.3-63.2) | 3.3 (2.9-3.7) |
| control | 1 | TRUE | 316 | 79.5 (78-81) | 48.1 (42.7-53.5) | 3.7 (3.3-4.1) |
| intervention | 0 | FALSE | 693 | 74 (71-75) | 41.3 (38-45.2) | 1.7 (1.5-1.8) |
| intervention | 0 | TRUE | 126 | 71 (65.5-75.5) | 42.1 (34.1-50) | 1.5 (1.2-1.9) |
| intervention | 1 | FALSE | 126 | 79 (78-82) | 46.8 (38.1-55.6) | 3.1 (2.5-3.7) |
| intervention | 1 | TRUE | 534 | 80 (79-80) | 46.6 (42.3-50.8) | 3.2 (3-3.5) |

```
comply_table_cats <- long_compliance %>%
  group_by(name) %>%
  summarise(n=n(),
            model_preference = ci_bootstrap(max_score - first_assigned,mean,print=T,pct=T),
            pct_model_prioritized = round(mean(max_score)*100,1),
            pct_dispatcher_prioritized = round(mean(first_assigned)*100,1),
            pct_max_news = ci_bootstrap(max_news,mean,print=T,pct=T)) %>%
  filter(n>50) %>%
  arrange(desc(n))

kable(comply_table_cats,
      caption = "Comparison of model and dispatcher preferences per call type")
```

Comparison of model and dispatcher preferences per call
type


| name | n | model\_preference | pct\_model\_prioritized | pct\_dispatcher\_prioritized | pct\_max\_news |
| --- | --- | --- | --- | --- | --- |
| Trauma | 641 | -8.9 (-12.6–4.8) | 31.5 | 40.4 | 47.7 (44-51.8) |
| Difficulty Breathing | 387 | 12.7 (7.7-17.6) | 71.8 | 59.2 | 70.3 (65.6-74.9) |
| Abdominal/flank pain | 266 | -9 (-16.2–1.9) | 35.3 | 44.4 | 49.2 (43.2-54.9) |
| General Elderly | 263 | 11.8 (6.1-17.9) | 62.7 | 51.0 | 55.9 (49.8-61.6) |
| Dizziness | 155 | -9.7 (-18.1–2.6) | 20.0 | 29.7 | 43.2 (36.1-51) |
| Infection | 137 | 21.2 (12.4-30.7) | 71.5 | 50.4 | 65 (56.9-73) |
| Back pain | 79 | -11.4 (-19–3.8) | 13.9 | 25.3 | 43 (31.6-54.4) |
| Arm/leg sympoms (non-traumatic) | 77 | -1.3 (-11.7-9.1) | 26.0 | 27.3 | 50.6 (39-62.3) |
| General Adult | 77 | 3.9 (-6.5-15.6) | 48.1 | 44.2 | 59.7 (49.4-71.4) |
| Fever | 75 | 22.7 (13.3-33.3) | 82.7 | 60.0 | 77.3 (68-86.7) |
| Fainting | 72 | -13.9 (-25–1.4) | 22.2 | 36.1 | 38.9 (29.1-50) |
| Stroke | 68 | -7.4 (-20.6-5.9) | 41.2 | 48.5 | 52.9 (41.2-64.7) |
| Cardiac arrythmia | 61 | 3.3 (-9.8-16.4) | 50.8 | 47.5 | 42.6 (31.1-55.7) |
| Chest pain | 61 | -16.4 (-31.2-1.6) | 32.8 | 49.2 | 60.7 (49.2-72.2) |

## Estimate accuracy using model assignments in high-confidence intervention cases

```
mi_long_hybrid <- complete(mi_obj,action = "long", include = TRUE) %>%
  # Do item level imputation https://www.tandfonline.com/doi/abs/10.1080/00273171.2012.640589
  select(-news_full) %>%
  mutate(news_full = rowSums(dplyr::select(., starts_with("news_")))) %>%
  mutate(out_primary = pmin(pmax(amb_airway,amb_breathing,amb_circulation,news_con),1),
         out_prio = pmin(pmax(amb_prio,amb_crit),1),
         out_intervention = pmin(pmax(amb_meds,amb_iv,news_o2,amb_alert),1),
         out_hosp = pmin(pmax(hosp_admit,hosp_30daymort),1),
         out_composite = out_primary*4+out_prio*2+out_intervention+out_hosp) %>%
  left_join(select(incl_data,id,TrialID,Score,conf),by = c("id","TrialID")) %>%
group_by(.imp,TrialID) %>%
  mutate(max_news = news_full == max(news_full),
         max_out = out_composite == max(out_composite,na.rm = T),
         first_assigned_hybrid = ifelse(Group == "intervention" & conf == "high" & !is.na(conf),
                                    max_score,first_assigned),
         news_diff = news_full[first_assigned_hybrid] - mean(news_full[!first_assigned_hybrid],na.rm=T)) %>%
  ungroup() %>%
  filter(as.logical(first_assigned_hybrid)) %>%
  select(-starts_with("cat"),-starts_with("mbs")) %>%
  mutate(conf = relevel(factor(conf),ref = "low"))
```

```
## Warning: There were 181 warnings in `mutate()`.
## The first warning was:
## ℹ In argument: `max_out = out_composite == max(out_composite, na.rm = T)`.
## ℹ In group 4: `.imp = 0` and `TrialID = "20210202195449_775475_775522_ap0"`.
## Caused by warning in `max()`:
## ! no non-missing arguments to max; returning -Inf
## ℹ Run `dplyr::last_dplyr_warnings()` to see the 180 remaining warnings.
```

```
mi_long_hybrid %>%
  filter(.imp != 0) %>%
  group_by(Group) %>%
  summarise(mean(max_news))
```

```
mi_test_hybrid <- mi_long_hybrid %>% as.mids()
```

```
## Warning: Number of logged events: 2
```

```
fit_test_hybrid <- with(mi_test_hybrid,
                    glm(max_news ~ Group,
                        family = "binomial"))

test_hybrid <- summary(pool(fit_test_hybrid),conf.int = TRUE)


kable(test_hybrid)
```

| term | estimate | std.error | statistic | df | p.value | 2.5 % | 97.5 % |
| --- | --- | --- | --- | --- | --- | --- | --- |
| (Intercept) | 0.5128840 | 0.0883736 | 5.803591 | 746.4882 | 0.0000000 | 0.3393937 | 0.6863743 |
| Groupintervention | 0.4242787 | 0.1282191 | 3.309013 | 496.8481 | 0.0010042 | 0.1723602 | 0.6761972 |

```
exp(select(test_hybrid,estimate,`2.5 %`,`97.5 %`))
```

## Parameter summary / variable importance

```
model_props <- fromJSON(file = "Study/data/model_props.json")

prop_table <- tibble(name = names(model_props$feat_props$median),
                     median = unlist(model_props$feat_props$median),
                     gain = unlist(model_props$feat_props$gain))

features <- tibble(name = model_props$feature_names) %>%
  left_join(prop_table) %>%
  mutate(gain = ifelse(is.na(gain),0,round(gain,1)),
         description = case_when(grepl("^disp_cat",name) ~ "CDSS category",
                                 grepl("^disp_q",name) ~ "CDSS question",
                                 grepl("^reg_",name) ~ "Call region",
                                 grepl("^text_",name) ~ "Freetext word/bigram",
                                 grepl("^disp_lat|^disp_lon",name) ~ "Call GPS location",
                                 grepl("^Priority",name) ~ "Call priority",
                                 grepl("^RecomendedPriority",name) ~ "CDSS recomended priority",
                                 grepl("^disp_age",name) ~ "Patient age",
                                 grepl("^disp_gender",name) ~ "Patient gender",
                                 name %in% c("disp_date","disp_hour","disp_month") ~ "Call date/time")) %>%
  select(description,everything()) %>%
  arrange(desc(gain))
```

```
## Joining with `by = join_by(name)`
```

```
datatable(features)
```

This table presents all parameters included in the models and the
average gain across the 4 outcome models for each included predictor.
Parameters with a gain of 0 were not included in any of the models. Note
that many parameter names are as they are shown in the CDSS in Swedish,
with non-ascii characters (ä/ö/å) removed.

## AUC in retrospective test and trial data

```
eval_trial <- mi_long_calib %>%
  filter(.imp == 1) %>%
  select(Score,out_primary,out_prio,out_intervention,out_hosp,out_composite) %>%
  mutate(type = "trial")

eval_test_labels <- read_csv("Study/data/labels.csv") %>%
  transmute(caseid,
            out_primary = amb_eval,
            out_prio = amb_prio,
            out_intervention = amb_intervention,
            out_hosp = hosp_care,
            out_composite = 4*amb_eval + 2*amb_prio,+amb_intervention + hosp_care)
```

```
## Rows: 192102 Columns: 5
## ── Column specification ────────────────────────────────────────────────────────
## Delimiter: ","
## chr (1): caseid
## dbl (4): amb_intervention, amb_prio, amb_eval, hosp_care
## 
## ℹ Use `spec()` to retrieve the full column specification for this data.
## ℹ Specify the column types or set `show_col_types = FALSE` to quiet this message.
```

```
eval_combined<- tibble(caseid = names(model_props$scores),
                    Score = unlist(model_props$scores)) %>%
  left_join(eval_test_labels) %>%
  mutate(type = "test") %>%
  bind_rows(eval_trial)
```

```
## Joining with `by = join_by(caseid)`
```

```
paste_auc_ci <- function(l,s,r = 3){
      ci <- round(as.numeric(ci(roc(l,s))),r)
      return(paste0(ci[2]," [",ci[1],",",ci[3],"]"))
}

aucs <- eval_combined %>%
  group_by(type) %>%
  summarise(auc_primary = paste_auc_ci(out_primary,Score),
            auc_prio = paste_auc_ci(out_prio,Score),
            auc_intervention = paste_auc_ci(out_intervention,Score),
            auc_hosp = paste_auc_ci(out_hosp,Score))
```

```
## Setting levels: control = 0, case = 1
## Setting direction: controls < cases
## Setting levels: control = 0, case = 1
## Setting direction: controls < cases
## Setting levels: control = 0, case = 1
## Setting direction: controls < cases
## Setting levels: control = 0, case = 1
## Setting direction: controls < cases
## Setting levels: control = 0, case = 1
## Setting direction: controls < cases
## Setting levels: control = 0, case = 1
## Setting direction: controls < cases
## Setting levels: control = 0, case = 1
## Setting direction: controls < cases
## Setting levels: control = 0, case = 1
## Setting direction: controls < cases
```

```
kable(aucs)
```

| type | auc\_primary | auc\_prio | auc\_intervention | auc\_hosp |
| --- | --- | --- | --- | --- |
| test | 0.746 [0.735,0.757] | 0.668 [0.653,0.682] | 0.583 [0.574,0.592] | 0.648 [0.639,0.657] |
| trial | 0.701 [0.677,0.726] | 0.652 [0.61,0.695] | 0.619 [0.598,0.64] | 0.687 [0.667,0.706] |

This table presents the area under a reciever operating
characteristics curve value for both a retrospective randomly held-out
test dataset, as well as corresponding values for predictions made
during the trial, for each of the 4 outcomes included in the composite
score with 95% DeLong Confidence intervals. Multiclass AUC values are
provided for the composite score, though confidence intervals are not
implemented for this type of AUC value.
