# Supplementary material for: Machine learning assisted differentiation of low acuity patients at dispatch: The MADLAD randomized controlled trial
Source: PLoS Med. 2026 Mar 31;23(3):e1004770. doi: 10.1371/journal.pmed.1004770 (PMC13037975; doi:10.1371/journal.pmed.1004770)
Supplement: S1 Study Protocol — (PDF) [file pmed.1004770.s002.pdf]

Study Title:

Machine Learning Assisted Differentiation of Low-Acuity Patients at Dispatch (MADLAD): A Randomized Controlled Trial

Document version: 1.2

Document date: 2021-02-09

Abstract

BACKGROUND:

At Emergency Medical Dispatch (EMD) centers, Resource Constrained Situations (RCS) where there are more callers requiring an ambulance than there are available ambulances are common. At the EMD centers in Uppsala and Västmanland, patients experiencing these situations are typically assigned a low-priority response, are often elderly, and have non-specific symptoms. Machine learning techniques offer a promising but largely untested approach to assessing risks among these patients.

OBJECTIVES:

To establish whether the provision of machine learning-based risk scores improves the ability of dispatchers to identify patients at high risk for deterioration in RCS.

DESIGN:

Multi-centre, parallel-grouped, randomized, analyst-blinded trial.

POPULATION:

Adult patients contacting the national emergency line (112), assessed by a dispatch nurse in Uppsala or Västmanland as requiring a low-priority ambulance response, and experiencing an RCS.

OUTCOMES:

Primary:

1. Proportion of RCS where the first available ambulance was dispatched to the patient with the highest National Early Warning Score (NEWS) score

Secondary:

- Difference in composite risk score consisting of ambulance interventions, emergent transport, hospital admission, intensive care, and mortality between patients receiving immediate vs. delayed ambulance response during RCS.
- Difference in NEWS between patients receiving immediate vs. delayed ambulance response during RCS.

INTERVENTION:

A machine learning model will estimate the risk associated with each patient involved in the RCS, and propose a patient to receive the available ambulance. In the intervention arm only, the assessment will be displayed in a user interface integrated into the dispatching system.

TRIAL SIZE:

1500 RCS each consisting of multiple patients randomized 1:1 to control and intervention arms

Table of Contents

Abstract 2

Version History: 4

Introduction 4

- Background 4
- Objectives 5
- Hypotheses 5

Methods 5

- Design 5
- Setting 5
- Eligibility criteria 6
- Flow 6
- Intervention 7
- Outcomes 7
- Participant timeline 8
- Sample Size 8
- Recruitment / Allocation 8
- Blinding 8
- Data management / Collection 8
- Statistical analysis 9
- Monitoring 10

Ethics 10

- Approval 10
- Consent 11
- Confidentiality 11
- Declaration of Interests 11
- Dissemination 11

References 11

Version History:

1.0 – Initial version (2020-09-14)

1.1 – Post pilot study (2021-01-25)

- Updated outcomes used in risk assessment models to include ambulance staff initial assessment results
- Simplified primary hypothesis to improve interpretability and take advantage of additional statistical power granted by improved prediction models. (proportion test vs. rank sum test)

1.2 – Post PRS review (2021-02-09)

- Add cover page

Introduction

Background

In many prehospital care systems, ambulance availability places constraints on the ability of Emergency Medical Dispatch Centers (EMDCs) to immediately provide an ambulance response for all patients determined to require one. The stochastic nature of ambulance demand via the emergency hotline (In Sweden, 112) entails that any cost-effective ambulance system will from time to time experience Resource Constrained Situations (RCS) in which the number of patients requiring an ambulance response exceeds the number of available ambulances.

In this study, we define an RCS as a situation in which EMDC staff must select one of multiple patients awaiting an ambulance to receive an ambulance response – i.e., the RCS is a discrete event occurring at the point in time at which a dispatch decision must be made. An RCS can arise either due to an absolute lack of available ambulances, or due to the need to maintain readiness to respond to high-priority patients. In some RCS, non-clinical factors such as relative geographical distances between patients and ambulances, or the amount of time patients have been waiting may determine the appropriate patient to dispatch an ambulance to. In many situations however, prioritization decisions are made by weighing the clinical condition of patients against each other. Given the heterogeneous nature of the patient population served by prehospital care providers, this can often be a difficult and complex decision.

In previous research, a machine learning-based risk assessment instrument was developed and validated retrospectively in a cohort of patients receiving prehospital care. (1) These instruments predict the likelihood of a patient experiencing a given set of clinical outcomes, such as hospital admission or mortality, and combining these likelihoods into an overall risk score. In this study, these tools will be applied to stratify patient risks, and serve as a decision support tool with the aim of ensuring that the patient with the most acute medical condition receives an immediate ambulance response in each RCS.

While a number of machine learning based risk assessment tools have been proposed for use in healthcare and validated retrospectively (2–4), there are few prospective trials, and none regarding models for risk stratification in broad patient cohorts in emergency care. There is thus a great need to identify suitable use cases for these tools, and to demonstrate their effectiveness in achieving clinically important objectives. The need for this evidence must however be balanced with the need to maintain a high degree of patient safety.

Objectives

This study aims to investigate whether the application of a machine learning-based risk assessment instrument improves the ability of dispatchers to identify and dispatch an ambulance to the most critically ill patient in an RCS. The criticality of the patient's condition will be operationalized primarily as the National Early Warning Score (NEWS) based on the first set of vital signs obtained by the ambulance, and secondarily based on a composite risk score consisting of prehospital interventions and hospital outcomes.

Hypotheses

Primary:

1. The intervention results in a greater proportion of immediate ambulance responses in RCS being directed to the patient in the most critical condition as operationalized by subsequent NEWS value.

Secondary:

1. The intervention improves differentiation with regards to a composite risk score consisting of ambulance interventions, abnormal initial ambulance findings, emergent transport, hospital admission, and mortality between patients receiving immediate vs. delayed ambulance response during RCS.
2. The intervention improves differentiation regarding NEWS between patients receiving immediate vs. delayed ambulance response during RCS.

Other pre-specified analyses:

1. Evaluation of overall personnel compliance with risk assessment instrument in intervention arm.
2. Evaluation of compliance in intervention arm cases where the model had a high vs low level of confidence.
3. Evaluation of improved/degraded compliance with risk assessment instrument over time as manifested by a slope change in a time series analysis of intervention group
4. Evaluation of spillover effects as manifested by a significant positive slope in a time series analysis of control group outcomes
5. Evaluation of change in risk assessment tool predictive value over time (covariate drift)
6. Evaluation of model calibration with regards to age, gender, and complaint category

Methods

Design

A parallel grouped trial, with groups randomized 1:1 to either the intervention or control arms using a random number generator-based procedure applied at the time of inclusion.

The unit of analysis in the study is the RCS, and outcome measures are based on the difference in score (NEWS or composite outcome score) between the patient receiving an immediate ambulance response, and patients receiving a delayed ambulance response per dispatch log data.

Setting

The study will take place in two EMDCs in central Sweden (Uppsala and Västmanland), serving a combined population of 499 000. The regions have a total of 32 ambulances during peak hours. Each dispatch center is staffed by 2-3 nurse call-takers and 1 ambulance director 24 hours per day. The

study has been piloted and initiated in the region of Uppsala, and Västmanland will join the study contingent on the collection of outcome data necessary to train and validate the risk assessment models.

In these regions during 2020, ca. 30% of callers received a high priority lights and sirens response, ca. 43% of patients received a low priority response, 5% were inter-facility transfers, and 22% of callers were referred to non-ambulance care. The median time from receipt of call to dispatch of ambulance (the "dispatch time") for low-priority ambulance responses was 18 minutes, and the median response time was 37 minutes. The dispatchers currently employ a self-developed, rule-based Clinical Decision Support System (CDSS) to structure patient interviews and determine a priority level, as described elsewhere (5,6).

Eligibility criteria

Inclusion Criteria:

- Identification of a resource constrained situation by ambulance director (i.e., 2 or more patients awaiting an ambulance response)
- Assigned priority 2A or 2B (Low-priority ambulance response) by dispatch nurse call-taker
- Valid Swedish personal identification number collected at dispatch
- Age >= 18 years

Exclusion Criteria

- Relevant calls received more than 30 minutes apart
- Logistical factors (eg. the patients' geographical locations) affect the ambulance assignment decision
- On scene risk factors (eg. a patient is outdoors and risks hypothermia) or risk mitigators (eg. healthcare staff already on-scene with a patient) affect the ambulance assignment decision

Flow

Prior to initiation of the study, the risk assessment tool will be integrated into the dispatching interface used by all study centers. Ambulance directors will have overall responsibility for executing the study protocol, and be tasked with identifying RCS suitable for inclusion in the study.

Patients are included in the study upon the identification of an RCS involving eligible patients by the ambulance director at the point in time when an ambulance is available for immediate dispatch to one of the patients. Directors are instructed to carefully consider any non-clinical factors relevant to prioritizing the patients prior to randomization, and exclude any RCS where these factors would override a clinical determination per the above exclusion criteria. Upon selecting the relevant patients and pressing a button in the interface to compare the selected patients, the RCS will be randomly assigned to a study arm. In the control arm, the risk scores for each patient will be calculated and stored, but not displayed to the user. In the intervention arm, a mark will be displayed in the interface indicating which of the included patients has the highest risk score, along with a color-coded indicator of model confidence.

In both study arms, the ambulance director will confer with the nurses involved in triaging the patients to confirm which patient should receive the available ambulance. In the intervention arm, the ambulance director will note which patient was proposed by the machine learning framework in conference with the nurses. The director will then dispatch the available ambulance to the patient determined through this process to have the greatest need. This process will be repeated each time an ambulance becomes available.

Intervention

The intervention is based on a risk assessment instrument validated in a previous study. (1) Since the publication of the validation study, the risk assessment instrument been further developed to include free-text notes entered by dispatchers, which were found to improve the performance of the models. The full source code of the tool employed in the study is freely available for replication purposes: https://github.com/dnspangler/openTriage.

The framework estimates the likelihood that a patient will be assessed by ambulance crews to 1) have abnormal initial findings, 2) be transported to the hospital with lights and sirens, 3) receive a prehospital intervention, and 4) be admitted to the hospital, or die within 30 days. Hospital outcome measures are based on the first hospital visit within 72 hours to capture hospital visits of non-conveyed patients. The predicted likelihood for each of the outcomes is then combined into a composite risk score, with the above outcomes weighted 4:2:1:1. In each RCS included in the intervention arm, the patient with the highest composite risk score will be indicated in a user interface integrated in the Alitis dispatching system used in Uppsala and Västmanland. A graphical indicator of model confidence will be integrated in the user interface, notifying users as to whether the model has a high- or low level of confidence in the prediction, with a cutoff value calibrated to include ca. 50% of patients in each group.

Development of the modelling framework will be frozen upon initiation of the main study phase. New predictor and outcome data will however continue to be captured and used to update the models in a quarterly monitoring and model updating process to ensure patient safety.

Outcomes

The primary outcome of the study is based on the National Early Warning Score (NEWS) of each included patient based on the first set of vital signs captured by the ambulance crew upon arrival to the patient. If Ambulance vitals signs are missing (e.g., if a patient opted to take an alternate mode of transport to the hospital), the first set of vital signs obtained at ED triage will be used. NEWS was selected as the primary outcome of the study for two reasons: Firstly, NEWS is widely used in acute care, and has been thoroughly validated as being predictive of outcomes in a variety of adult patient cohorts.(7–10) Secondly, NEWS is based on patient vital signs, and is thus conceptually distinct from-, and prior in terms of causality to the outcome measures we employ to train the predictive models.

The latter reason is a subtle but important conceptual point which addresses two issues: Firstly, by selecting an evaluation measure which is not causally dependent on (or indeed identical to) the outcomes used to train the models, we minimize the possibility that assignment to the intervention or control arm in and of itself affects the evaluation measure (e.g., if it were communicated to the ambulance crew that a patient had a high risk of transport to the hospital using lights and sirens, this could influence the decisions of the crew, perhaps making them more likely to do so). Secondly, it addresses issues relating to AI system alignment. As suggested by the orthogonality thesis, the predictive performance of an AI system is independent of the goals of the system as a whole. (11) Operationalizing the need for a rapid ambulance response in terms of measurable outcomes is difficult, and we cannot assume that we have done so perfectly. Thus, it is appropriate that both the model and human decisions are evaluated in terms of a measure which in causally independent of either. In this way, we avoid giving the ML framework an unfair advantage over human dispatchers who may have internalized a different definition of patent risk and ambulance care need.

The first secondary outcome in the study is based on a composite score consisting of each of the four outcomes included in the risk assessment instrument. The outcomes will be weighted in a manner identical to the prediction framework. While this measure of intervention effectiveness suffers from

the problems noted above, this is the manner in which predictive models are typically evaluated.(3,4) Evaluating a measure of performance in terms of the goals the system was designed to achieve is thus also considered appropriate.

Participant timeline

All outcome data are extracted algorithmically from ambulance and hospital databases, and the intervention occurs at a single point in time during the handling of the patient's call at the EMDC. As such, no follow up assessments involving the patient are required.

Sample Size

Given the prevalence and diversity of non-clinical determinants in resolving RCS, the actual retrospective accuracy of dispatchers in identifying the most critically ill patients could not be determined. Instead, sample size was determined using a simulation-based approach. Low-priority calls are divided into 2 categories – 2A and 2B. A simulation was performed under the assumption that a priority 2A call would always be selected for immediate ambulance dispatch over a priority 2B call, and that dispatchers would have 100% compliance with the machine learning tool.

Based on 1000 bootstrap resampled pairs of patients ranked using either dispatch category or the ML framework, a U-statistic of 0.545 in favor of the ML framework was identified. This resulted in required sample size of 1500 at α= 0.05, β = 0.85, and a randomization ratio of 1:1 for a Wilcoxon–Mann–Whitney test of difference in central tendency. (12) Based on an estimate of 2 RCS per day meeting all inclusion criteria, an upper bound on the data collection duration was set to 2 years.

Upon further refinement of the prediction models over the course of the pilot study, it was found that this difference-based test had an excess of power, and a more conceptually simple but less sensitive formulation of the primary hypotheses was adopted. Pilot study data indicate that RNs direct the available ambulance to the patient with the highest NEWS score 63.5% of the time (considering ties as "correct" assessments), while simulations suggest that the model will mark the patient with the highest NEWS correctly in 70.3% of cases, resulting in an estimated power of 0.8 at n=1500 using a two-sided test of proportions.

Recruitment / Allocation

Patients will be recruited by ambulance directors upon identification of an RCS. Study arm allocation will be performed automatically by the server used to generate risk assessments using a simple random number generator implemented by the numpy python package.

Blinding

Patients and ambulance crews will be blind to treatment arm allocation, but by the nature of the intervention, dispatchers will be aware of the randomization results. In extracting study data for final analysis, treatment group allocation will be assigned a randomized code to blind the analyst to treatment group assignment. All analyses will furthermore be performed using code made available for peer review and published along with the manuscript and will, insofar as possible, be written prior to final data extraction.

Data management / Collection

Data collected by EMDCs regarding dispatcher decisions, machine learning model predictions, treatment group allocation, etc., will be stored in the currently employed dispatch system database used by both Uppsala and Västmanland to limit exposure of patient data. Database queries will be performed against this database, and combined with ambulance and hospital data collected in an

existing database used for quality assurance/improvement at Region Uppsala. Similar queries will be developed to extract ambulance and hospital data from Region Västmanland, and the need to develop these data extraction processes entails that Västmanland will begin including patients to the study after Region Uppsala. Manual data entry will be performed only to correct errors, and random spot-checks of data quality will be performed. These processes will be similar to those employed to generate the data used in the retrospective study validating the methods used here. (1)

Statistical analysis

To generate risk predictions, gradient boosting models are applied to patient demographics, structured CDSS data, and free-text noted embedded using the bag-of-words method. The methods are implemented in the openTriage platform as the 'uppsala_alitis' framework (13) and have been validated in previous research.

The primary hypotheses will be evaluated using a standard two-sided test of proportions between the intervention and control groups. Investigating the secondary hypotheses will involve comparing outcomes for the patient receiving an ambulance immediately with patients for whom dispatch is delayed. Patients who do not receive an ambulance immediately may also potentially be included in later trials. This induces a potential that information from intervention arm comparisons could be used by dispatchers in control arm comparisons, thus biasing the results in the direction of a smaller intervention effect. Steps were taken in the development of the user interface to limit these "spillover" effects.

Figure 1 – Analysis flow

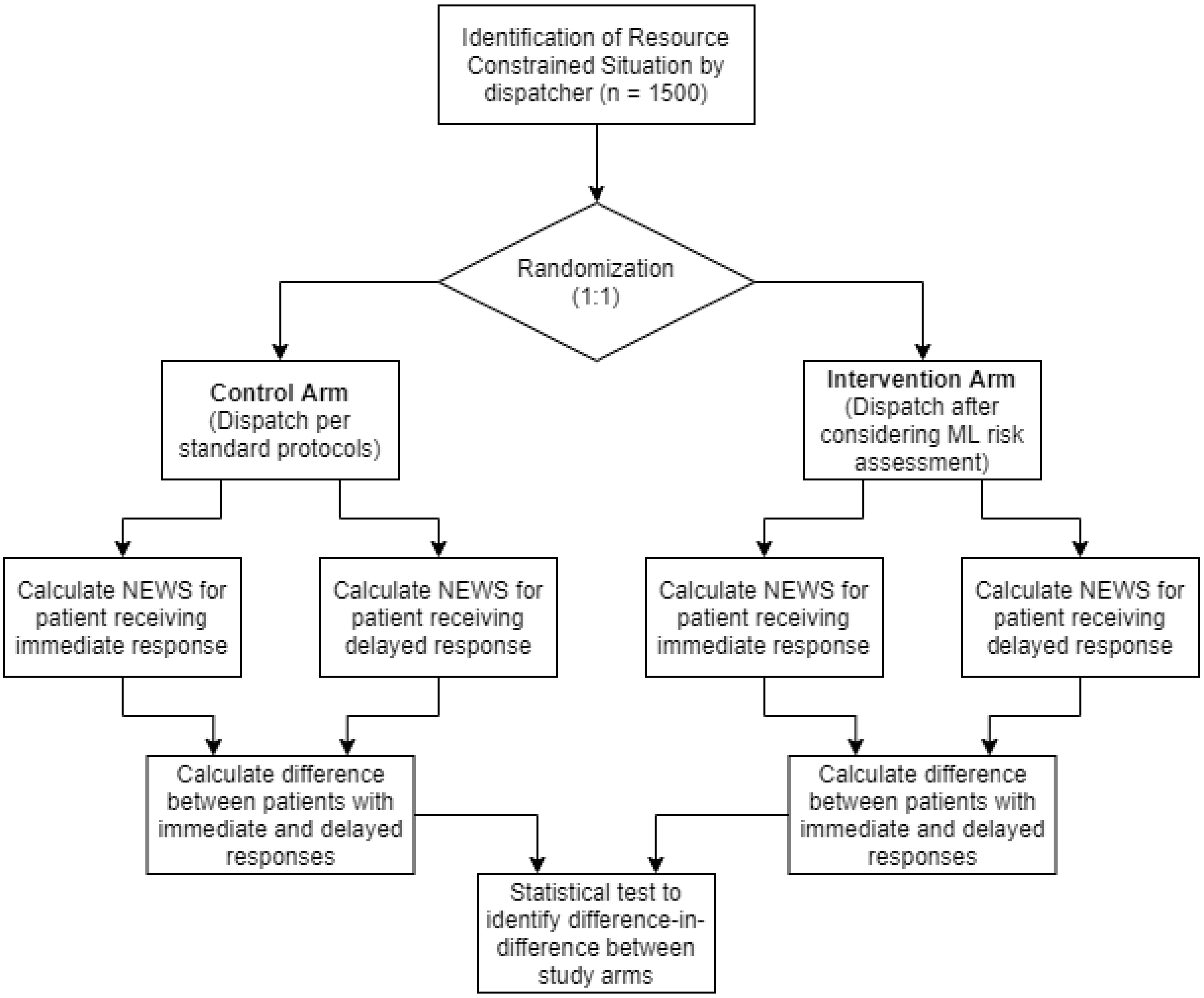

Additional pre-specified analyses 1-2 will be investigated by assessing the compliance of dispatchers with the risk assessment tool of risk scores in the high and low confidence groups of the intervention arm (calibrated based on pilot study data to consist of ca. 50% of assessments each), as well as dispatcher compliance, with the hypothesis that compliance will be higher in the high-confidence group.

Pre-specified analyses 3-5 will be examined using time-series analysis within a regression framework employing a variable representing the study month as the independent variable of interest. Analysis 6 will be conducted by constructing calibration curves for the risk model based on the patient groups of interest.

Missing vital sign data will be imputed using Multivariate imputation by chained equations. (14) As we found previously that between-imputation variance after calculating NEWS scores was minimal, the median of 5 imputed NEWS values will be used to assess the primary hypothesis. An analysis of loss to follow-up (i.e., patients requesting to withdraw from the study) will be performed.

Monitoring

All dispatch system data will be collected within a single IT infrastructure under the direct supervision of the PI. Outcome data will be collected either directly by the research group, or in close cooperation with the research group in the case of Västmanland. Randomly sampled outcome data will be manually checked to ensure quality. The research group will conduct regular follow-ups with dispatchers responsible for executing the study protocol to ensure compliance.

A data monitoring committee (DMC) consisting of the medical directorship of the ambulance services and dispatch centers in the two studied counties will handle adverse events (AEs) and make decisions regarding early trial stoppage. Early stopping will be evaluated quarterly based on the Haybittle–Peto rule, with a p-value of <0.01 bounding an effect regarding the primary hypothesis. (15) If this threshold is reached, the treatment group will be unblinded to determine if the effect is detrimental, in which case the study will be halted to ensure patient safety. No early stopping rule for a beneficial effect will be employed.

Theoretical model performance will be evaluated as part of the quarterly DMC reviews. If theoretical model performance is found to suffer from a significant reduction in performance during a given month, hyperparameter optimization will be attempted. If theoretical model performance degrades beyond the point at which a clinical benefit from the intervention can reasonably be expected, a decision by the DMC to pause the trial until the issues can be corrected may be taken. Any changes to model hyperparameters or the estimation framework will be noted in the manuscript, and be made available for peer- and public review.

The existing adverse event reporting system in both counties will be monitored by the DMC for AEs relating to the patients included in the trial. Any AEs reported by patients or providers will be evaluated by the DMC.

Ethics

Approval

Ethical approval for the study was sought and granted by the Swedish Ethical Review Authority (dnr 2020-00187). The study will be conducted in accordance with the Helsinki declaration and relevant Swedish law.

Consent

An exemption from gathering prospective informed consent from patients was granted for the study by the ethics review board. Informed consent materials will be mailed to study participants retroactively, at which point patients may withdraw from the study.

Confidentiality

Data will be handled in accordance with the EU General Data Protection Regulations and relevant Swedish law. All identifiable patient data will be handled by employees of Region Uppsala with extant access to the data, and only researchers at Uppsala University will have access to de-identified research datasets. Access to the research data will be provided to third party researchers after publication upon reasonable request, and the research group will undertake to develop an anonymized version of dataset for public release.

Declaration of Interests

The researchers declare no competing interests. The tools investigated in this trial are provided under open-source licenses to the public free of charge.

Dissemination

The study results will be published in a peer-reviewed, open-access journal. Authorship eligibility will be based on ICMJE recommendations. The protocol will be provided along with the study, along with all statistical analysis code necessary to replicate the results.

References

1. Spangler D, Hermansson T, Smekal D, Blomberg H. A validation of machine learning-based risk scores in the prehospital setting. Ashkenazi I, editor. PLOS ONE. 2019 Dec;14(12):e0226518.

2. Blomberg SN, Folke F, Ersbøll AK, Christensen HC, Torp-Pedersen C, Sayre MR, et al. Machine learning as a supportive tool to recognize cardiac arrest in emergency calls. Resuscitation. 2019 May;138:322–9.

3. Levin S, Toerper M, Hamrock E, Hinson JS, Barnes S, Gardner H, et al. Machine-Learning-Based Electronic Triage More Accurately Differentiates Patients With Respect to Clinical Outcomes Compared With the Emergency Severity Index. Ann Emerg Med. 2018 May 1;71(5):565-574.e2.

4. Hong WS, Haimovich AD, Taylor RA. Predicting hospital admission at emergency department triage using machine learning. PLOS ONE. 2018 Jul 20;13(7):e0201016.

5. Spangler D, Edmark L, Winblad U, Colldén-Benneck J, Borg H, Blomberg H. Using trigger tools to identify triage errors by ambulance dispatch nurses in Sweden: an observational study. BMJ Open. 2020 Mar 1;10(3):e035004.

6. Holmström IK, Kaminsky E, Lindberg Y, Spangler D, Winblad U. Registered Nurses' experiences of using a clinical decision support system for triage of emergency calls: A qualitative interview study. J Adv Nurs. 2020;76(11):3104–12.

7. Brangan E, Banks J, Brant H, Pullyblank A, Roux HL, Redwood S. Using the National Early Warning Score (NEWS) outside acute hospital settings: a qualitative study of staff experiences in the West of England. BMJ Open. 2018 Oct 1;8(10):e022528.

8. Pimentel MAF, Redfern OC, Gerry S, Collins GS, Malycha J, Prytherch D, et al. A comparison of the ability of the National Early Warning Score and the National Early Warning Score 2 to identify patients at risk of in-hospital mortality: A multi-centre database study. Resuscitation. 2019 Jan 1;134:147–56.

9. Silcock DJ, Corfield AR, Gowens PA, Rooney KD. Validation of the National Early Warning Score in the prehospital setting. Resuscitation. 2015 Apr 1;89:31–5.

10. Pirneskoski J, Kuisma M, Olkkola KT, Nurmi J. Prehospital National Early Warning Score predicts early mortality. Acta Anaesthesiol Scand. 2019;63(5):676–83.

11. Bostrom N. The superintelligent will: Motivation and instrumental rationality in advanced artificial agents. Minds Mach. 2012;22(2):71–85.

12. Shieh G, Jan S, Randles RH. On power and sample size determinations for the Wilcoxon–Mann–Whitney test. J Nonparametric Stat. 2006 Jan 1;18(1):33–43.

13. Spangler D. openTriage [Internet]. 2020 [cited 2020 Jun 25]. Available from: https://github.com/dnspangler/openTriage

14. Buuren S van, Groothuis-Oudshoorn K. Multivariate Imputation by Chained Equations in R. J Stat Softw [Internet]. 2011 Dec 12 [cited 2017 May 4];45(3). Available from: https://www.jstatsoft.org/article/view/v045i03

15. Pocock SJ. When to stop a clinical trial. BMJ. 1992 Jul 25;305(6847):235–40.
