# Supplementary material for: Machine learning assisted differentiation of low acuity patients at dispatch: The MADLAD randomized controlled trial
Source: PLoS Med. 2026 Mar 31;23(3):e1004770. doi: 10.1371/journal.pmed.1004770 (PMC13037975; doi:10.1371/journal.pmed.1004770)
Supplement: S1 CONSERVE Checklist — ‌‌ (DOCX) [file pmed.1004770.s004.docx]

# CONSERVE Checklists

Use CONSERVE-CONSORT for completed trial reports and CONSERVE-SPIRIT for trial protocols.

| CONSERVE-CONSORT Extension: 2026-02-18 | | | | | |
| --- | --- | --- | --- | --- | --- |
| Item | Item Title | Description | | | Section, paragraph no. |
| I. | Extenuating Circumstances | Describe the circumstances and how they constitute extenuating circumstances. | | | (see below) |
| II. | Important Modifications | 1. Describe how the modifications are important modifications. | | | (see below) |
|  |  | 1. Describe the impacts and mitigating strategies, including their rationale and implications for the trial. | | | (see below) |
|  |  | 1. Provide a modification timeline. | | | (see below) |
| III. | Responsible Parties | State who planned, reviewed and approved the modifications. | | | (see below) |
| IV. | Interim data | If modifications were informed by trial data, describe how the interim data were used, including whether they were examined by study group, and whether the individuals reviewing the data were blinded to the treatment allocation. | | | (see below) |
| CONSORT Number and Item | | For each row, if important modifications occurred check “direct impact” and/or “mitigating strategy” and describe the changes in the trial manuscript or supplement. Check “no change” for items that are unaffected in the extenuating circumstance. | | | Section, paragraph no. |
|  |  | No Change | Impact* | Mitigating Strategy** |  |
| 1 | Title and abstract | X |  |  |  |
| 2 | Introduction | X |  |  |  |
| 3 | Methods: Trial Design | X |  |  |  |
| 4 | Methods: Participants |  |  | X | Participants, 1 |
| 5 | Methods: Interventions |  |  | X | Machine learning model, 3 |
| 6 | Methods: Outcomes | X |  |  |  |
| 7 | Methods: Sample Size | X |  |  |  |
| 8-10 | Methods: Randomisation | X |  |  |  |
| 11 | Methods: Blinding | X |  |  |  |
| 12 | Methods: Statistical methods |  |  | X | Statistical analysis, 1 |
| 13 | Results: Participant flow | X |  |  |  |
| 14 | Results: Recruitment | X |  |  |  |
| 15 | Results: Baseline data | X |  |  |  |
| 16 | Results: Numbers analysed | X |  |  |  |
| 17 | Results: Outcomes and estimation |  |  |  |  |
| 18 | Results: Ancillary analyses | X |  |  |  |
| 19 | Results: Harms | X |  |  |  |
| 20 | Discussion: Limitations | X |  |  |  |
| 21 | Discussion: Generalisability | X |  |  |  |
| 23 | Other information: Registration |  |  |  |  |
| 24 | Other information: Protocol | X |  |  |  |
| 25 | Other information: Funding | X |  |  |  |
| *Aspects of the trial that are directly affected or changed by the extenuating circumstance and are not under the control of investigators, sponsor or funder.  **Aspects of the trial that are modified by the study investigators, sponsor or funder to respond to the extenuating circumstance or manage the direct impacts on the trial. | | | | | |
